# Diagnosis, treatment, and misdiagnosis analysis of 28 cases of central nervous system echinococcosis

**DOI:** 10.1186/s41016-021-00248-y

**Published:** 2021-05-21

**Authors:** Guojia Du, Yandong Li, Pan Wu, Xin Wang, Riqing Su, Yandong Fan, Dangmurenjiafu Geng

**Affiliations:** Department of Neurosurgery, The First Teaching Hospital of Xinjiang Medical University, Xinjiang, 830054 China

**Keywords:** Echinococcosis, Central nervous system, Diagnosis, Treatment

## Abstract

**Background:**

To explore central nervous system (CNS) involvement in this disease, from the perspectives of diagnosis, treatment, and misdiagnosis

**Methods:**

Twenty-eight patients with CNS echinococcosis were included in this retrospective study, including 18 males (64.3%) and 10 (35.7%) females. The average age of all the patients were 23.5 years (ranged 460 years). Twenty-three (23) patients (82.1%) received the first surgical resection in our hospital. Five (5) patients (17.9%) gave up surgical treatment for multiple-organ hydatidosis and previous surgery history at other hospitals, and albendazole was applied for a long-term (36 months) adjunct therapy for the 5 patients. The average follow-up time was 8 years.

**Results:**

For the 28 patients, 23 cases received surgical treatments, and the diagnosis was confirmed by pathological examinations. The diagnosis of 4 cases of brain echinococcosis and 2 cases of spinal cord echinococcosis could not be confirmed, resulting in a misdiagnosis rate of 21.4% (6/28). For the pathological examination, a total of 17 cases were infected with *Echinococcus granulosus* (including 2 cases of spinal cord echinococcosis), and 6 cases were infected with *Echinococcus alveolaris*.

**Conclusion:**

The diagnosis should be specifically considered in endemic regions. The clinical features of CNS hydatidosis were intracranial space-occupying lesions. For the treatment, the surgical removal of cysts should be necessary. In addition, the adjuvant therapy with drug and intraoperative prophylaxis is also suggested. The misdiagnosis may have resulted from atypical clinical features and radiographic manifestations, as well as the accuracy of hydatid immunologic test.

## Background

Echinococcosis is a zoonosis with global prevalence, which is caused by the larval stage of the *Echinococcus* tapeworm. There are mainly two types of hydatid disease, caused by *Echinococcus granulosus* and *Echinococcus alveolaris*. *Echinococcus alveolaris*-caused echinococcosis is less common but more invasive compared to *Echinococcus granulosus*, presented features like malignancy. Echinococcosis is prevalent in the developing countries, and it is generally observed in the great grazing regions in the world, such as Australia, New Zealand, Mongolia, Japan, and Indonesia. Echinococcosis is also prevalent in China, especially in western regions without adequate sanitation, such as Xinjiang, Ningxia, Inner Mongolia, and Qinghai.

The diagnosis and treatment of echinococcosis have been a significant issue that attracted attentions from both physicians and researchers. Echinococcosis can infect multiple organs of humans, resulting in complications during the long course of diseases. Here, a retrospective study was performed on 28 cases of CNS echinococcosis treated in our hospital during January 2000 and May 2019. These patients were analyzed from the perspectives of diagnosis, treatment, and misdiagnosis reasons. The results were further discussed with literature.

## Methods

### Demographic data

A total of 28 patients were admitted in our hospital for echinococcosis, including 26 cases of brain echinococcosis and 2 cases of spinal cord echinococcosis. There were 18 (18/28, 64.3%) males and 10 (10/28, 35.7%) females. The average age was 23.5 years, ranging from 4 to 60 years. The average course of disease before hospitalization was 6 months, ranging from 1 month to 3 years. For the ethnicity, there were 12 cases of Han, 5 cases of Kazak, 4 cases of Mongolian, 4 cases of Uygur, 2 cases of Tibetan, and 1 case of Hui. Liver and lung infection of hydatid cysts was concomitantly observed in 10 patients; 5 cases received the treatment of surgery in other hospital and then presented with relapse and metastasis (Table 1).

**Table 1:** Summary of 28 cases of central nervous system echinococcosis

NO.	Year	Age	Sex	Cyst location	Other cyst locations	Preoperative complaints	Surgery (yes/no)	Pathological examination	Misdiagnosis (yes/no)
1	2000	60	M	Multiple	Kidney, liver	Headache, nausea and vomiting, seizure, consciousness disturbance	No	No	No
2	2000	23	F	Right parietal	Liver	Headache, vision loss	Yes	EA	Yes
3	2000	21	M	Left temporal	Liver, lung	Headache, seizure	Yes	EG	No
4	2000	16	M	Right temporal	Kidney	Headache	Yes	EG	Yes
5	2001	22	F	Multiple	Liver, Lung	Consciousness disturbance	No	No	No
6	2001	12	M	Multiple	None	Headache, nausea and vomiting, diplopia	Yes	EG	Yes
7	2002	24	M	Multiple	Liver, lung	Consciousness disturbance	No	No	No
8	2003	4	M	C5-T2	None	Stiff neck and limb weakness	Yes	EG	Yes
9	2003	39	M	Right cerebellum	Liver, lung	Consciousness disturbance	No	No	No
10	2004	45	M	L5-S1	None	Urinary incontinence, lower limbs weakness	Yes	EG	Yes
11	2006	39	F	Multiple	Spleen, liver	Headache, nausea and vomiting	Yes	EG	No
12	2006	15	F	Multiple	Liver, lung	Headache, nausea and vomiting	Yes	EG	No
13	2006	18	M	Multiple	None	Headache, nausea and vomiting	Yes	EG	Yes
14	2007	27	F	Left cerebellum	Liver, lung	Headache, nausea and vomiting, ataxia, stiff neck	Yes	EG	No
15	2007	24	F	Right cerebellum	Heart	Headache, stiff neck, ataxia	Yes	EG	No
16	2007	17	F	Multiple	None	Headache, consciousness disturbance	Yes	EG	No
17	2008	24	M	Multiple	Liver, lung	Consciousness disturbance	No	No	No
18	2008	18	F	Left parietal	None	Headache, diplopia, vision loss	Yes	EG	No
19	2009	11	M	Left frontal	None	Headache	Yes	EG	No
20	2009	21	F	Left parietal	Liver, lung	Headache, nausea and vomiting, diplopia, vision loss	Yes	EA	No
21	2010	16	M	Right temporal	Liver, lung	Headache, seizure	Yes	EG	No
22	2010	31	F	Left frontal	None	Headache, nausea and vomiting	Yes	EG	No
23	2012	38	M	Multiple	None	Headache, nausea and vomiting, vision loss	Yes	EG	No
24	2014	18	M	Multiple	Liver, lung	Headache, ataxia, vision loss	Yes	EA	No
25	2016	20	M	lateral ventricle	None	Headache	Yes	EG	No
26	2016	12	M	Multiple	None	Consciousness disturbance	Yes	EA	No
27	2018	26	M	Multiple	Liver	Headache, nausea and vomiting, vision loss	Yes	EA	No
28	2019	20	M	Multiple	None	Headache, nausea and vomiting	Yes	EA	No

*M* Male, *F* Female, *EG Echinococcus granulosus*, *EA Echinococcus alveolaris*

### Clinical manifestations

For the clinical features, there were 21 cases of headache, 14 cases of nausea and vomiting, 7 cases of consciousness disturbance, 17 cases of papillary edema, 6 cases of vision loss, 4 cases of diplopia, 9 cases of visual field defects, 5 cases of limb paresthesia, and 7 cases of muscle weakness. Cerebral hernia was observed in 2 patients, stiff neck was observed in 3 patients, epilepsy was observed in 5 patients, and unilateral paralysis was observed in 2 patients. Pathological signs were positive in 14 patients, nystagmus was observed in 4 patients, and ataxia was observed in 4 patients.

### Imaging examination

**Fig. 1:**
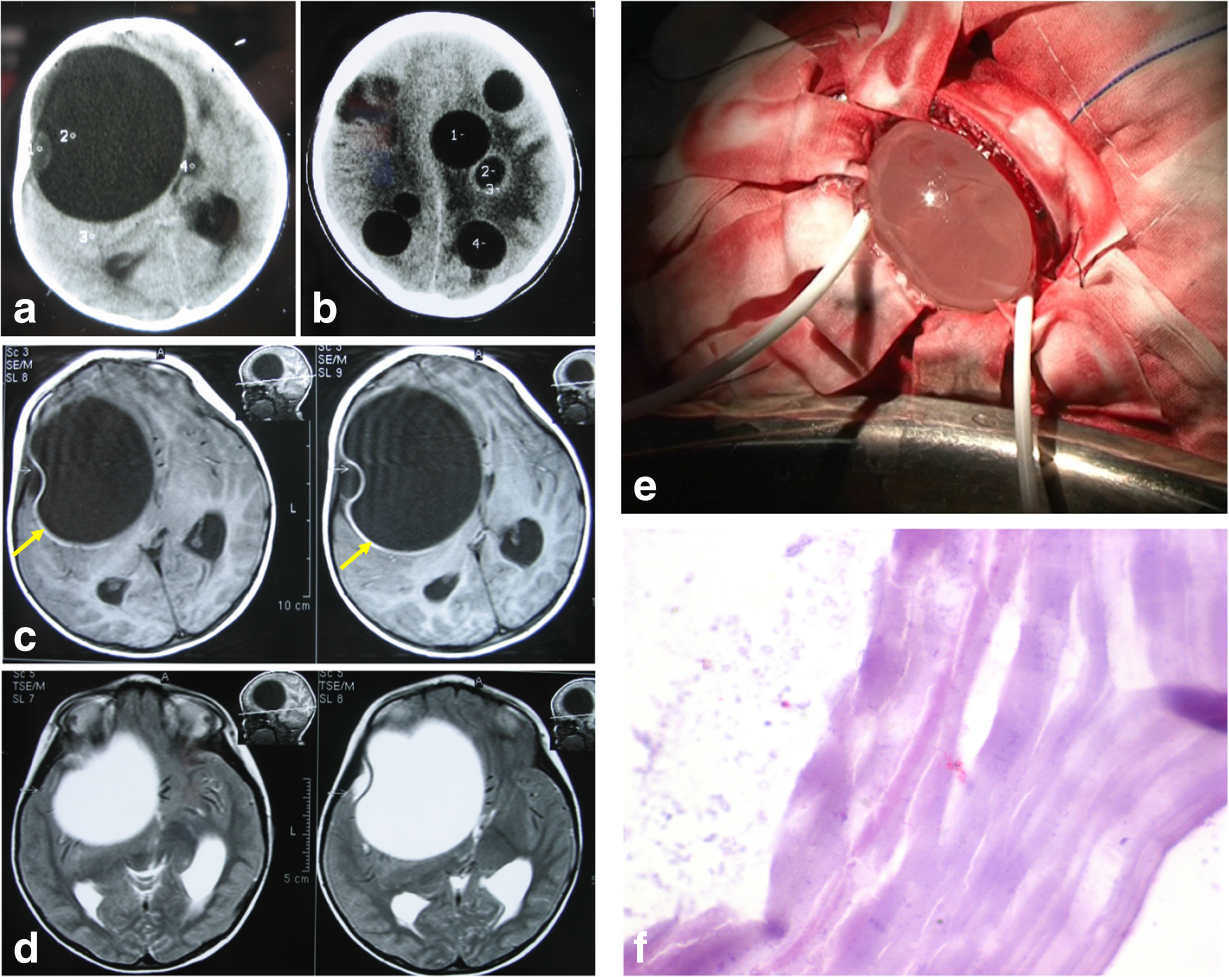
Brain *Echinococcus granulosus*. **a** CT scan revealing a solitary cystic lesion located in the right frontotemporal lobe. The margins are well delineated. The intralesional fluid displays the same attenuation value as that of CSF. There is no obviously perilesional edema. **b** CT scan shows multiple cystic lesions in frontoparietal lobe, with the same density as CSF. **c** MRI T1-weighted images shows a homogeneous, thin-walled cystic lesion (yellow arrows). There is significant mass effect on the lateral ventricular system. Midline shift is 2 cm. Cyst signal is isointense relative to CSF. **d** MRI shows a cystic lesion hyperintense on T2-weighted images (similar to CSF) with a hypointense halo around the cyst capsule (yellow arrow). **e** Dowlings technique. **f** Photomicrography (HE, original magnification, 400) shows *Echinococcus granulosus* infection of the brain

**Fig. 2:**
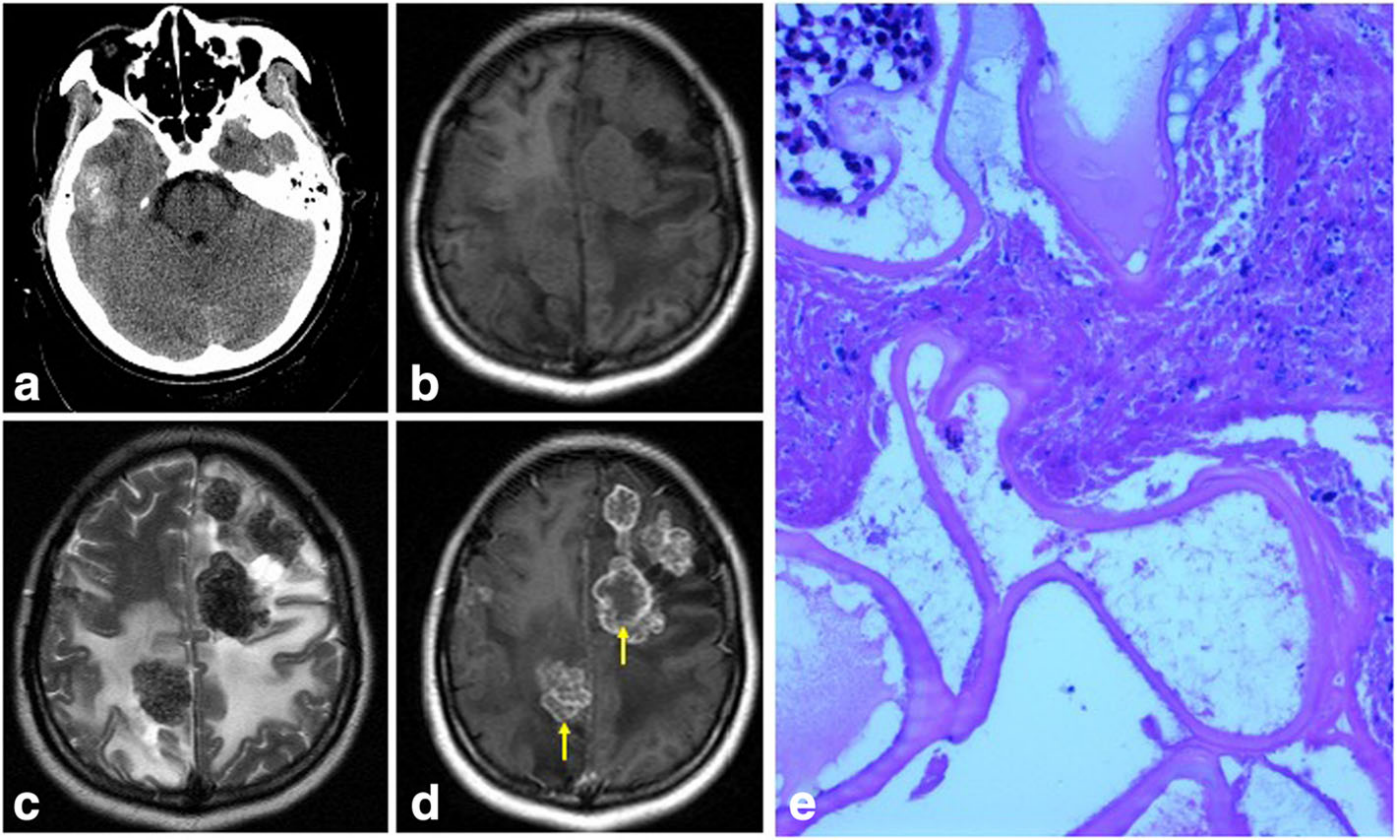
Brain *Echinococcus alveolaris*. **a** CT scan shows a mixed density solid lesion located in the right temporal lobe. There is slight perilesional edema. **b** MRI T1W axial MR image shows multiple equal T1 signal intensity lesion located in frontoparietal lobe. There is obviously perilesional edema. **c** MRI T2W axial MR image shows a short T2 signal intensity multiple lesions with obviously perilesional edema located in the same place. **d** Contrast-enhanced MR image shows obvious enhancement of the lesion wall (yellow arrows). **e** Photomicrography (HE, original magnification, 400) shows *Echinococcus alveolaris* infection of the brain

Supratentorial and subtentorial hydatidosis was observed in 22 and 4 patients, respectively. Spinal cord hydatidosis was observed in 2 patients. For the intracranial hydatidosis, multiple lesions were found in 14 patients, and single lesion was observed in 12 patients (2 cases of frontal, 3 cases of parietal lobe, 3 cases of temporal lobe, 1 case of lateral ventricle, and 3 cases of cerebellum). The diameter of hydatid cyst was from 13 to 85 mm. Computed tomography (CT) and magnetic resonance imaging (MRI) were performed for all the 28 patients (Figs. 1ad and Fig. 2ad).

### Laboratory tests

Hydatid immunologic test was performed for all the patients. During 20002005, 10 cases were tested with routine 3-item hydatid immunologic test (Casonis method, indirect blood coagulation method, counter immune electrophoresis), and during 20062019, 18 cases were tested with 8-item immunodiagnostic kit, namely, immunogold filtration assay and enzyme-linked immunosorbent assay (ELISA) for testing four hydatid antigens, including capsule liquid antigen (EgCF), head quarter antigen (EgP), sac liquid half purification antigen (EgB), and alveolar hydatid cyst antigen (Em2).

### Treatment

Twenty-three patients (23/28, 82.1%) received surgery. The surgical plans (including surgical approaches, bone choice, and dural window) were designed according to the sites and size of the lesions, as well as the maximum diameter of the cyst. Dowlings technique was applied in 17 patients to remove the cyst wall from the surrounding brain parenchyma with irrigation (Fig. 1e). The hydatid with surrounding edema was completely excised in 6 patients. Five (5/28, 17.9%) patients gave up surgery due to multiple-organ hydatidosis and history of previous surgery at other hospitals, and then, they received a long-term adjunct therapy of albendazole, with the dose of 15 mg/kg/d PO divided bid for 3 to 6 months.

## Results

Preoperatively, 22 cases were diagnosed as brain echinococcosis based on the combination of clinical manifestations, hydatid immunologic test, and imaging examination. Six cases were not diagnosed as echinococcosis, including 2 cases of spinal cord echinococcosis and 4 cases of brain echinococcosis, with a misdiagnosis rate of 21.4% (6/28). For the single hydatid immunologic test, 10 cases received routine 3-item hydatid immunologic test during 20002005, and there were 7 positive cases, with a positive rate of 70.0%; 18 cases received hydatid 8-test immunodiagnosis during 20062019, and there were 15 positive cases, with a positive rate of 83.3%.

Twenty-three patients (23/28, 82.1%) received surgical resection. Postoperatively, the pathological examinations showed 17 cases of *Echinococcus granulosus* (Fig. 1f) (including 2 cases of spinal cord echinococcosis) and 6 cases of alveolar *Echinococcus* (Fig. 2e). Another 5 patients (5/28, 17.9%) gave up surgical treatment and received a long-term drug therapy of albendazole (36 months).

The average follow-up period was 8 years, ranging from 3 months to 11 years. For the 17 patients who received Dowlings technique to dissect the cyst, no postoperative recurrence was reported. Four patients experienced relapse and another operation within 6 months to 1 year. Two patients died of intracranial infection and cachexia. For the 5 patients who received drug therapy, 4 patients suddenly died of cerebral hernia within 34 years. One patient is still alive.

## Discussion

Hydatid disease has been widely prevalent in the developing countries, and it is both a medical and economic issue. Echinococcosis was generally rare in developed countries; however, it has still been a significant public health issue in endemic areas [1,3]. The prevalence of echinococcosis is also observed in western China. Echinococcosis is one of the most common zoonosis, which equally infects both genders. The younger populations were subjected to more serious infections [4]. The definite hosts of *Echinococcus* are a variety of carnivores, and dogs are the most common host. Sheep, cattle, and human beings can act as the intermediate host.

The transmission routes of echinococcosis are through contacting with definite host or consuming contaminated water or food. During the infection, the eggs of *Echinococcus* lose their enveloping layer in the stomach, and the embryos would be released. The embryos pass through the wall of the gut into the portal system, being transferred to the liver. Most larvae would be entrapped end encysted in the liver. Some larvae may occasionally reach the lungs, and some may enter the systemic circulation after passing through the capillary filter of the liver and lungs. Some of these larvae may reach the brain or spinal cord. Brain echinococcosis are often accompanied with liver and lung infections. In our study, 16 of the 28 patients showed hydatid cysts in other organs. For the cerebral hydatid cysts, most cases were supratentorial, while only a few were infratentorial lesions [5]. Brain hydatid can be rarely located in some special place, such as thalamus or ventricle [6, 7]. Intracranial hydatid cysts were generally solitary, while multiple cysts were rare [8, 9]. Intracranial hydatid cyst may also be classified as primary or secondary infection. The primary cysts resulted from direct infestation of the larvae in the brain, without significant involvement of other organs. The secondary multiple cysts resulted from spontaneous, traumatic, or surgical rupture of the primary intracranial hydatid cyst, which may lack brood capsule and scolices. In our study, the primary cyst was observed in 11 cases, and the secondary multiple cysts were found in 10 cases.

Hydatid disease of the spinal cord has been rare. The first case of spinal cord echinococcosis was reported in 1964, in Xinjiang. Notably, approximately 50% of the patients were under 30 years old [10,15]. Braithwaite and Lees [16] have classified this disease into 5 types: (1) primary intramedullary hydatid cyst, (2) intradural extramedullary hydatid cyst, (3) extradural intraspinal hydatid cyst, (4) hydatid disease of the vertebra, and (5) paraspinal hydatid disease. Based on this classification, the 2 cases of spinal cord echinococcosis in our study were classified as type 3, the extradural intraspinal hydatid cyst.

The clinical manifestations of echinococcosis are similar to those of increased intracranial pressure due to other causes. The early diagnosis of echinococcosis was difficult for those patients without apparently increased intracranial pressure [17]. Therefore, a detailed medical history record would be especially important, and it should be combined with an auxiliary examination. Attention should be paid for the following situations: (1) the patient comes from epidemic area, being exposed to patients in these regions, or reporting contact with cattle and pet; (2) the patients can be asymptomatic or show elevated intracranial pressure, epilepsy, and nervous system local functional defect; (3) imaging examinations may reveal characteristic findings of brain hydatid cysts, especially head CT and MRI [18,21]; (4) the diagnosis may be supported by positive hydatid immunological test, but cannot be excluded by negative result; (5) the exclusion of intracranial space-occupying lesions due to other causes is important; the diagnosis of intracranial cystic brain echinococcosis should be differentiated from intracranial arachnoid cysts, brain-perforating deformity, and brain abscesses. The MRI result can be enhanced for intracranial bubbly brain echinococcosis, which can be distinguished from intracranial strengthened TB tumor, glioma, brain metastases, and brain cysticercosis. In addition, spinal cord echinococcosis should be differentiated from spinal cord and arachnoid cyst, as well as hydatid vascular malformation, such as spinal cord bleeding.

The diagnosis of echinococcosis should be comprehensively made based on patient information, clinical features, imaging results, and laboratory data. The clinical features may not be obvious. The imaging results could be atypical and similar to those of other intracranial space-occupying lesions; the differential diagnosis would be difficult. The radiologists from non-epidemic area may lack experience in recognizing the characteristic echinococcosis images. The diagnosis may be missed without adequate information and laboratory data. Therefore, the good communication between radiologists and clinician is necessary. Even so, there were still false-positive results [22], and such misdiagnosis may result from cyst wall thickness, deep location, and small number of cysts. Misdiagnosis of parasites may also be observed in organs with physical barriers, like the brain. The laboratory data may be affected in immunodeficiency and immunocompromised populations, including children, people undergoing chemotherapy, patients with tumor cachexia, and AIDS patients, which may also lead to misdiagnosis.

Until now, echinococcosis could still not be cured with any drug. The early diagnosis and timely treatment are critical for controlling brain echinococcosis. The preferred therapy is surgery. The preoperative plan is important for the complete removal of hydatid lesions, such as accurate localization, proper surgical approach, and adequate bone window. The critical point for the treatment of *Echinococcus granulosus* was the removal of unruptured cyst. Once the cysts ruptured and entered the subarachnoid space, there would be widespread dissemination of scolices, leading to severe inflammation and anaphylactic reactions caused by the fluids containing numerous antigens. Dowling-Orlandos technique has been widely applied in the removal of intraparenchymal cysts [23]. The cyst would be exposed by a wide incision, and saline irrigation was performed with a rubber catheter from an inclined position of the head. In our study, Dowlings technique was conducted in 17 cases, and the cyst wall was removed from the surrounding brain parenchyma with irrigation. The cysts could be completely excised, and the prognosis was good. As an adjunct for therapy, albendazole was given to treat patients with multiple-organ involvement or gave up surgical treatment. The dose was 15 mg/kg/d PO divided into twice daily for 36 months.

## Conclusions

Echinococcosis can infect multiple organs of humans. The diagnosis should be specifically considered in endemic regions. The clinical features of CNS hydatidosis were intracranial space-occupying lesions. For the treatment, the surgical removal of cysts should be necessary. In addition, the adjuvant therapy with drug and intraoperative prophylaxis are also suggested. The misdiagnosis may have resulted from atypical clinical features and radiographic manifestations, as well as the accuracy of hydatid immunologic test.

## Data Availability

The datasets used and/or analyzed during the current study are available from the corresponding author on reasonable request.
